# The application of polypropylene mesh for testicular prosthesis in surgical castration for patients with prostate cancer

**DOI:** 10.1186/s12957-019-1709-2

**Published:** 2019-10-07

**Authors:** Yao-Hui Xia, Wei Huang, Chun-Xiao Yu, Bo Kong, Rui Qin, Peng-Fei Wang, Jie An, Yong-Qiang Xia

**Affiliations:** 1 Department of Urology, Women and Children’s Hospital of Linyi City, NO.1 Qinghe South Road, Luozhuang District Linyi, 276000 China; 2 Department of Urology, Central Hospital of Zao Zhuang Mineral Group, No.52 of Qilianshan Road, Xuecheng District, Zaozhuang, 277800 China

**Keywords:** Prostate cancer, Surgical castration, Polypropylene mesh for testicular prosthesis, Testicular prosthesis

## Abstract

**Background:**

To explore whether a polypropylene mesh is suitable for application as a new material for testicular prostheses.

**Methods:**

The data of 65 patients with advanced prostate cancer who underwent surgical castration in hospital were collected and analyzed. Patients who preferred to undergo traditional orchidectomy (*n* = 16) were assigned to the control group, and patients who underwent subcapsular orchiectomy plus implantation of a polypropylene mesh testicular prosthesis (*n* = 49) were assigned to the experimental group. The presence of hematoma, infection, and other complications in patients in these two groups were investigated at 3 and 12 months following the surgery. The patients were also followed up using a self-designed testicular castration satisfaction questionnaire.

**Results:**

A higher score indicated greater satisfaction. The mean score was 15.33 ± 2.85 in the experimental group and 4.63 ± 1.45 in the control group at 3 months after the surgery. The mean score was 14.92 ± 1.74 in the experimental group and 4.25 ± 1.61 in the control group at 12 months after the surgery. The difference between the two groups was statistically significant at the two time points (*P* < 0.01).

**Conclusions:**

Compared with orchidectomy alone, patients were more satisfied with subcapsular orchiectomy plus the implantation of a polypropylene mesh testicular prosthesis for the treatment of advanced prostate cancer. Furthermore, the polypropylene mesh testicular prosthesis maintained its original character over the duration of the study, with a good long-term effect. Thus, implantation of a polypropylene mesh testicular prosthesis is indicated to be safe and effective, and polypropylene mesh is potentially useful as a new material for testicular prostheses.

## Introduction

Studies have revealed that the incidence of prostate cancer is becoming significantly higher in men > 60 year old [1, 2]. Among these patients, some patients lose the chance of radical surgery due to serious cardiovascular disease, pulmonary dysfunction, and other diseases [3]. Some patients refuse simple endocrine therapy due to heavy economic burden and are afraid of the inferiority complex when facing orchidectomy. Hence, this segment of patients often gives up the opportunity of treatment. To date, China could not produce qualified testicular prostheses due to histocompatibility, while imported testicular prostheses are expensive. Furthermore, the problem of infection and other complications remains unresolved. Therefore, very few patients undergo testicular prosthesis implantation after surgical castration, which severely limits the number of patients who received this treatment [4, 5]. Therefore, patients with advanced prostate cancer from January 2011, who require surgical castration in our hospital, underwent the pilot treatment of subalbugineous testicular evisceration plus the implantation of polypropylene mesh testicular prostheses using a self-designed testicular prosthesis. The histocompatibility of this prosthesis was observed, the satisfaction of these patients was followed-up using a self-designed testicular castration satisfaction questionnaire, and the scores of these patients were recorded and statistically analyzed. By comparing with traditional orchidectomy, the safety and effectiveness of the implantation of the polypropylene mesh testicular prosthesis were evaluated, and it was determined whether a polypropylene mesh could be used as a new material for testicular prosthesis. Patients who preferred to undergo traditional orchidectomy were assigned to the control group. The histocompatibility and the satisfaction following surgical castration of the two groups of patients were compared. Details are reported as follows.

## Methods

### General data

Data of 65 patients with advanced prostate cancer, who underwent surgical castration in our hospital from January 2011 to March 2016, were collected and analyzed. Based on their preferences for the treatment options, 49 patients underwent subalbugineous testicular evisceration plus implantation of polypropylene mesh testicular prosthesis and were assigned into the experimental group. The mean age of these patients was 76.1 ± 4.5 years old, and mean preoperative serum prostate-specific antigen (PSA) levels were 49.3 ± 18.2 μg/ml. Furthermore, among these patients, 7 patients were in stage T3 and 11 patients was in stage T4. Bone scan revealed bone metastases in 11 patients. In addition, in terms of Gleason scores, 9 patients had ≤ 6 points, 13 patients had 7 points, and 27 patients had ≥ 8 points. In the control group, 16 patients received bilateral orchidectomy. The mean age of these patients was 75.6 ± 6.6 years old, and the mean preoperative PSA was 53.9 ± 20.4 μg/ml. Furthermore, among these patients, 3 patients were in stage T3 and 5 patients were in stage T4. Bone scan revealed bone metastases in 2 patients. In terms of Gleason scores, 4 patients had ≤ 6 points, 3 patients had score of 7 points, and 9 patients had ≥ 8 points. Statistical analysis revealed that differences in age, preoperative PSA, tumor stage, and Gleason score between the experimental group and control group were not statistically significant (Table 1).

**Table 1:** Demographics and perioperative data of patients

		Control group (*n* = 16)	Experimental group (*n* = 65)
Mean age (years)		75.6 ± 6.6	76.1 ± 4.5
PSA level (μg/ml)		53.9 ± 20.4	49.3 ± 18.2
Stages of cancer (*n*)	Stage T3	7	3
	Stage T4	11	5
Bone metastases (*n*)		11	2
Gleason scores (*n*)	≤ 6 points	9	4
	7 points	13	3
	≥ 8 points	27	9

#### Production and details of the polypropylene mesh testicular prosthesis

The testicular prosthesis was made of a polypropylene mesh provided by China Holycon Medical Supplies Co. Ltd. (Nantong, Jiangsu; patent name: testicular prosthesis, patent no.: ZL 201320681687.0, patent owner: Yongqiang Xia). This prosthesis was used in clinical practice after the approval of the Ethics Committee of the Central Hospital of Zaozhuang Mining Group of Shandong Province. Families of the patients were informed that the polypropylene mesh testicular prosthesis is not a testicular prosthesis certified by the China Food and Drug Administration, but a scrotum filling material made of medical materials, and the purpose of the implantation is to maintain the male self-esteem. Written informed consent was obtained from all patients.

### Surgical methods

#### Surgical method for the experimental group

**Fig. 1:**
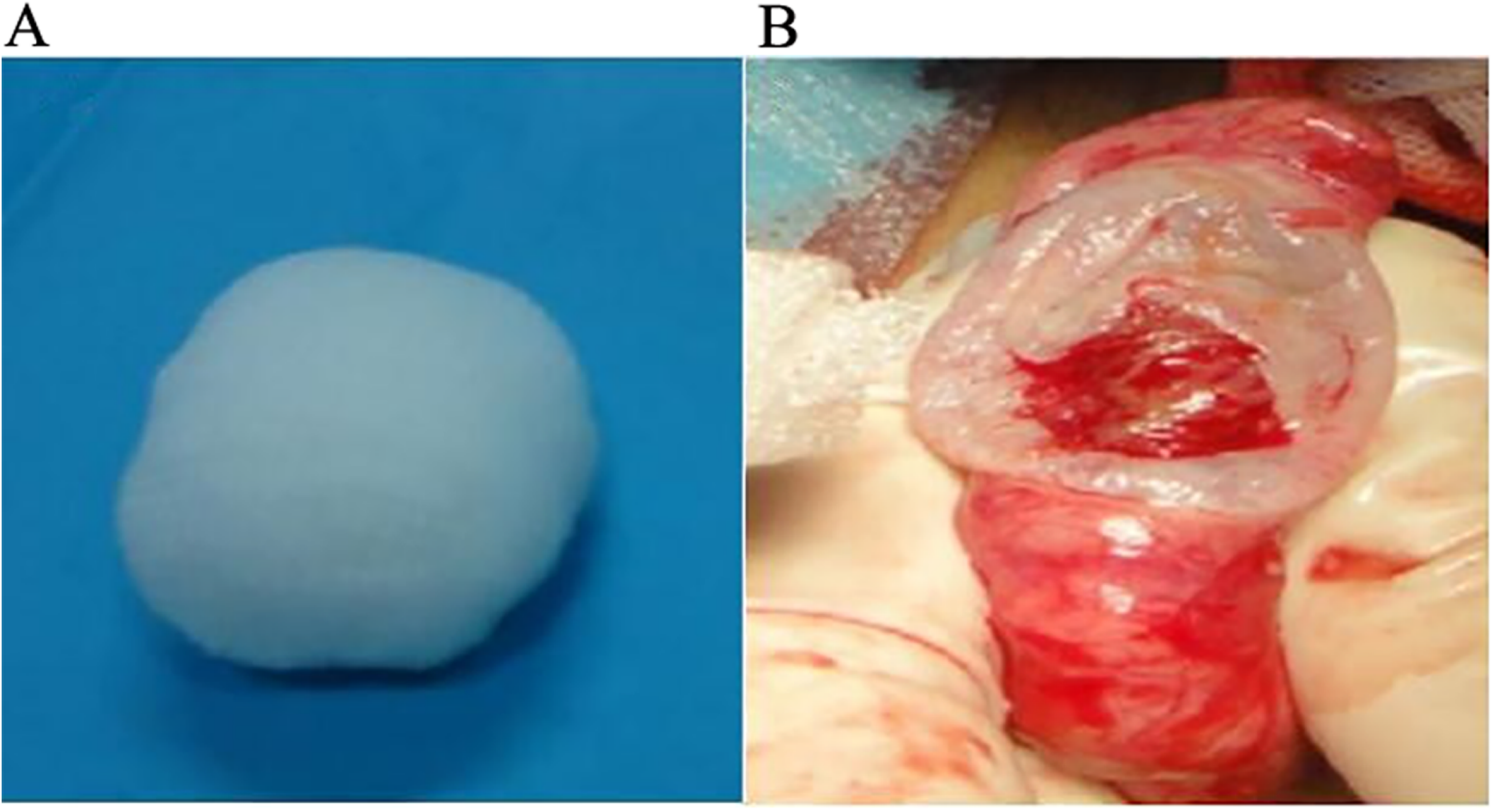
Testicular prosthesis (**a**) and orchiectomy (**b**)

**Fig. 2:**
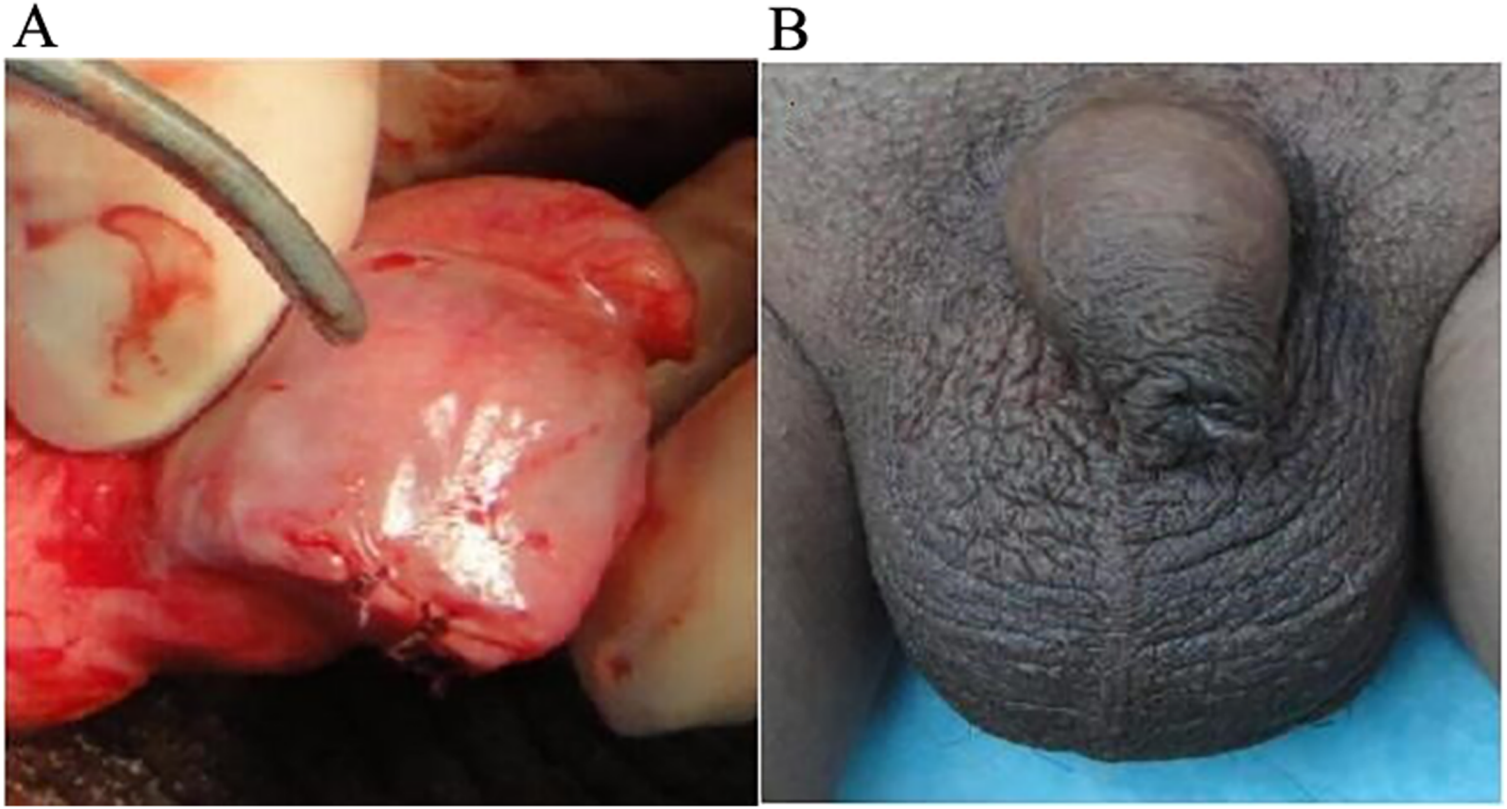
Testicular prosthesis implantation (**a**) and scrotal appearance after implantation (**b**)

Patients were laid in the supine position. General disinfection was performed, and surgical drapes were placed on the lower abdomen and scrotum. After the success of systemic or local anesthesia, palpation was performed to determine the size, shape, and texture of the testis and epididymis. The testis was adjusted to facilitate its free exterior edge to be the operative field, and the epididymis was placed in the deep part of the testis. The epididymis and testis were fixed by the left hand of the surgeon, while the assistant helped tighten the skin of the scrotum, in order to expose the incision of each layer. The skin and dartos coat were cut by the surgeon with a knife using his right hand. Next, the fascia spermatica externa, cremaster muscles, fascia spermatica interna, and tunica vaginalis of parietal layer were cut off layer by layer using an electrotome. Thus, the vaginal cavity was exposed, hydrocele and accessory calcium salt were removed, and iodophor disinfection was performed when necessary. Finally, the tunica vaginalis of the visceral layer and tunica albuginea were cut off. The polypropylene mesh testicular prostheses were previously produced with appropriate shapes. The contents of the testis were removed with a scoring tooth or a surgical knife handle. When obvious active bleeding occurred on the inner surface of the tunica albuginea, only appropriate electrocautery was conducted, in order to protect the blood supply of the tunica albuginea. The pre-made polypropylene mesh testicular prosthesis was implanted, and the tunica albuginea was continuously sutured with absorbable 4–0 suture. The testicular prosthesis and epididymis were adjusted to a suitable position, and two to three radial incisions were made on the tunica vaginalis of the wall layer with an electric knife, where the testis was located in the center of the incisions. Next, adequate hemostasis was performed. Interrupted suture was performed on the fascia spermatica externa, cremasteric muscle, and fascia spermatica interna (with three stitches each). The skin of the scrotum and dartos coat was sutured for three stitches for each layer. No drainage was placed in the incisions, while incisions were not treated with pressurized bandage. The contralateral implantation was treated in the same way. The appearance of the testicular prosthesis is shown in Fig. 1a. The appearance after testicular evisceration is shown in Fig. 1b. The appearance of the testis after the tamponage of testicular prosthesis is shown in Fig. 2a. In addition, the appearance of the scrotum after testicular prosthesis implantation is shown in Fig. 2b.

#### Operation method in the control group

The surgical procedures applied were the same as that in traditional orchiectomy.

### Evaluation method

The presence of hematoma, infection, and other complications in patients in these 2 groups were observed at 3 months and 12 months after the operation. The occurrence of prosthesis piercing the skin and autoimmune diseases were observed in the experimental group. A testicular castration satisfaction questionnaire was developed according to the concerned problems of patients and in reference to Kiyak’s satisfaction questionnaire [6] (Table 2). The scores of the 2 groups of patients were investigated at the above 2 time points using this questionnaire. Part of the patients failed to complete the 2 reviews and the 2 questionnaires due to lost to follow-up. At 3 months after the operation, 49 copies of questionnaires were completed in the experimental group, while 16 copies were completed in the control group. At 12 months after the operation, 45 copies of questionnaire were completed in the experimental group, while 15 copies were completed in the control group.

**Table 2:** The score distribution diagram of satisfaction of surgical castration

Observation item	Specific evaluation of each patient in two groups				
	2 points	1 point	0 point	− 1 point	− 2 point
1. Appearance of scrotum	Quite satisfied	Satisfied	Common	Dissatisfied	Quite dissatisfied
2. Size of scrotum	Quite satisfied	Satisfied	Common	Dissatisfied	Quite dissatisfied
3. Rigidity of scrotum	Quite satisfied	Satisfied	Common	Dissatisfied	Quite dissatisfied
4. Special discomfort of scrotum	None	Not obvious	A little	Obvious	Quite obvious
5. Degree of self-respect	Quite satisfied	Satisfied	Common	Dissatisfied	Quite dissatisfied
6. Adverse reactions	None	Not serious	Common	Serious	Quite serious
7. Feeling of invasive manipulation	Quite satisfied	Satisfied	Common	Dissatisfied	Quite dissatisfied
8. Effectiveness evaluation of household	Quite satisfied	Satisfied	Common	Dissatisfied	Quite dissatisfied
9. Impact of other factors (financial condition)	Quite small	Small	Common	Big	Quite big
10. Would like to recommend to friends?	Quite willing	Willing	Common	Unwillingness	Quite unwillingness

### Scoring method

Data in each item in the questionnaire were statistically processed. In the 5 columns of specific evaluation, the scores from left to right were 2 points, 1 point, 0 points, − 1 point, and − 2 points. Next, the average of the original scores was calculated and assigned as the satisfaction score. The higher the satisfaction score is, the higher the satisfaction degree of the patients on the operation is. The satisfaction scores between groups were compared using the Student *t* test.

## Result

**Fig. 3:**
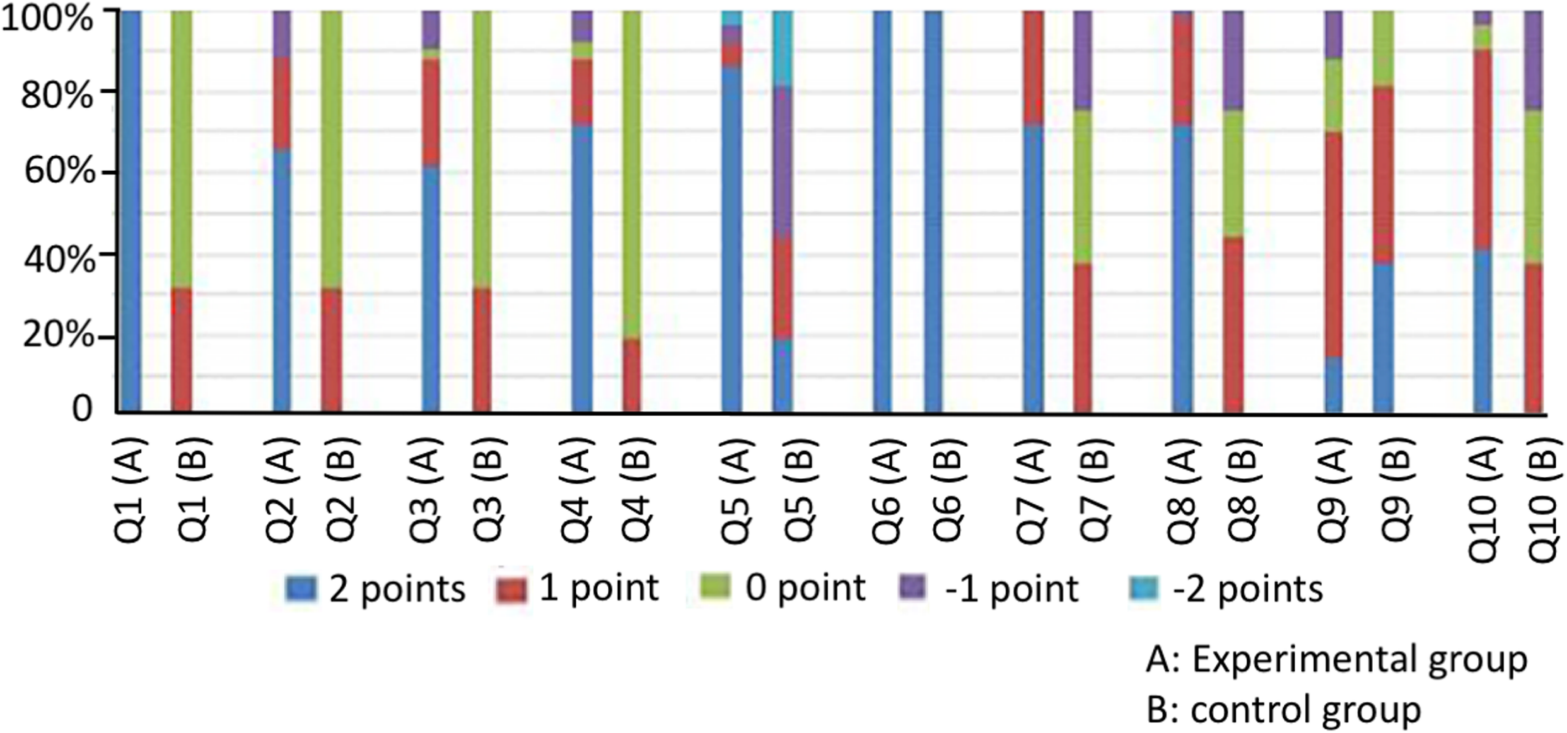
The score distribution diagram of satisfaction of testicular prosthesis 3 months after surgery. A: experimental group; B: control group; Q: question in the questionnaire

**Fig. 4:**
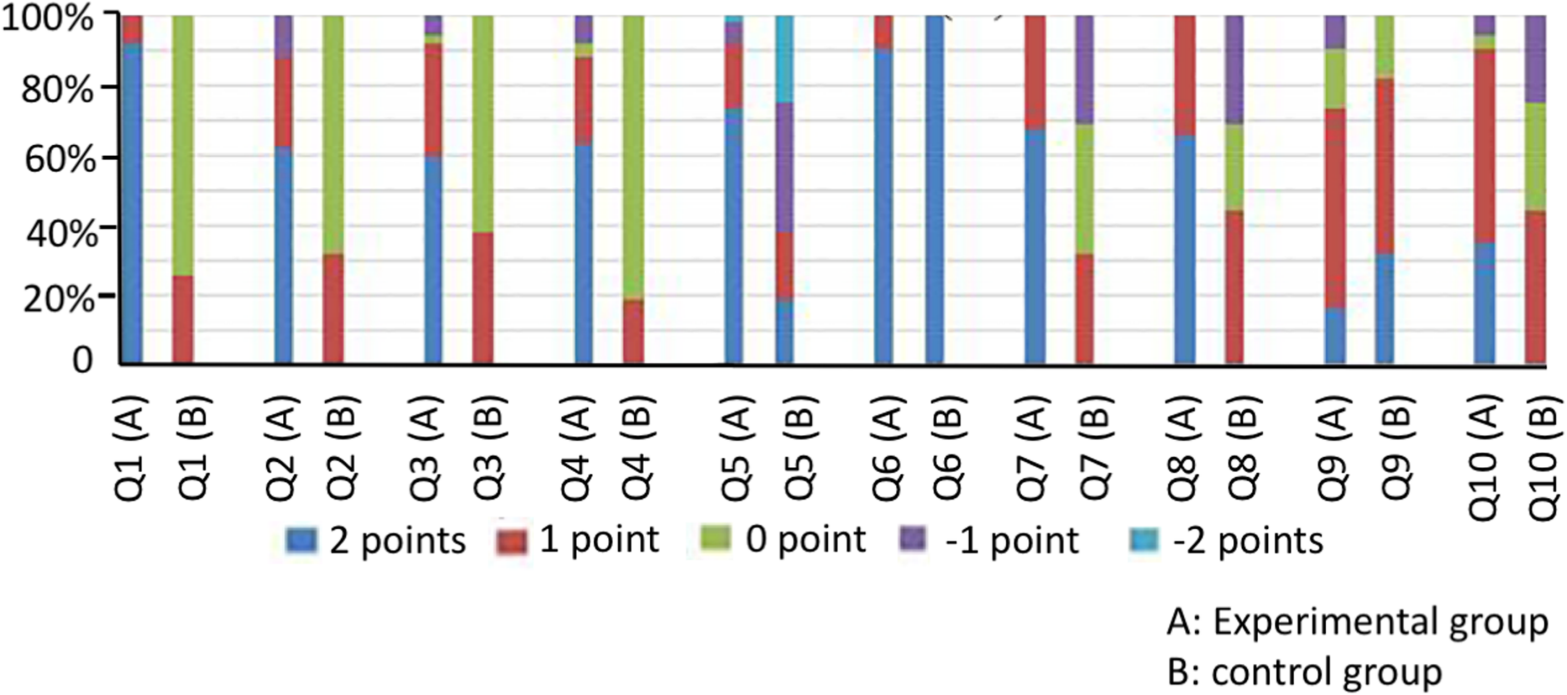
The score distribution diagram of satisfaction of testicular prosthesis 12 months after surgery. A: experimental group; B: control group; Q: question in the questionnaire

At 3 months and 12 months after the operation, no scrotum hematoma, infection, and other complications occurred in the two groups. No prosthesis pierced the skin in the experimental group, and no autoimmune disease was found (Figs. 3 and 4 and Table 3).

**Table 3:** The comparison of score distribution diagram for the satisfaction of surgical castration ($\overline{x}$ ± *S*)

Groups	Scores at 3 months after operation	Scores at 12 months after operation	*P* value
Experimental group	15.33 ± 2.85	14.92 ± 1.74	> 0.05
Control group	4.63 ± 1.45	4.25 ± 1.61	> 0.05
*P* value	< 0.01	< 0.01	

The satisfaction score was significantly higher in the experimental group than in the control group, and the difference between these two groups was statistically significant (*P* < 0.01). At 3 months and 12 months after the operation, intra-group differences within both the experimental group and control group were not statistically significant (*P* > 0.05). No complications induced by prosthesis were found in patients in the experimental group after being followed up for 1–5 years. However, statistical processing of satisfaction scores on this item was not conducted due to incomplete data.

## Discussion

In the global scale, the incidence of prostate cancer ranks second in all malignant tumors in men. Its incidence continues to increase year by year in China [7]. Since most prostate cancers are androgen-dependent and surgical castration, namely removal of the testes, can decrease serum testosterone by 95%, the lesions and symptoms of prostate cancer can be significantly alleviated. Androgen is secreted from interstitial cells in the testis. These types of cells are located in the testicular parenchyma. Therefore, subalbugineous testicular evisceration is a feasible way of surgical castration for patients with prostate cancer.

Orchidectomy has a close correlation to the quality of life of patients with prostate cancer. This is one of the important reasons for the decline in the quality of life of patients and has different degrees of influence on the physical function, role function, emotional function, cognitive function, and social function of patients [8–10]. This requires that more attention should be given to the adverse effects of orchidectomy on patients. Determining how to maximally reduce and avoid the influence of the quality of life of patients remains an urgent problem to be solved in clinical practice. Therefore, the implantation of a testicular prosthesis after castration would be a good remedy for prostate cancer patients, which is particularly important to reshape the appearance of men. Some studies found that the implantation of testicular prosthesis could significantly improve the self-image of patients undergoing orchidectom y[11]. Previously, testicular prostheses include Co-Cr-Mo alloy testicular prosthesis, synthetic resin testicular prosthesis, glass bead testicular prosthesis, dacron testicular prosthesis, polyethylene testicular prosthesis, silica gel testicular prosthesis [12], and tissue engineering cartilage testicular prosthesis [13]. However, the shortcomings of these prostheses include expensive price and susceptibility to postoperative complications such as pain, discomfort, scrotum hematoma, incision infections, and contracture of fibrous capsule around the prosthesis after surgery [14], which limits the development of the implanted testicular prosthesis.

Studies have confirmed that connective tissue in-grew into the meshes and formed an integrated and stable fibrous capsule after the implantation of polypropylene meshes, which reveal that the polypropylene mesh had a good histocompatibility [15]. Medical products made of polypropylene mesh have been widely used in pelvic floor reconstruction, hernia repair, and other fields, which have obtained good clinical results [15–17].

Prior to this present study, there are no related reports on polypropylene mesh testicular prosthesis at home and abroad [18]. Since the quantification standard of the effect after orchidectomy and testicular prosthesis implantation could not be searched, and in order to evaluate the application value of the implantation of the polypropylene mesh testicular prosthesis for prostate cancer patients undergoing surgical castration, the investigator for this study developed an observation questionnaire of testicular surgery using the following three items: the sense of the testis size, the sense of testicular rigidity, and the special indisposition of the testis. Most patients in the control group had a score of zero, since they recognized the objective condition of the loss of testis before surgery. However, the problem on whether the “testis” was good or bad was not present in these patients. Moreover, two patients considered that there were no bad to lose testis; thus, a score of 1 point was given. In the survey item “the impact of economic factors and other factors,” the scores were lower in the experimental group than in the control group. In the remaining survey items, the scores were higher in the experimental group than in the control group. This study found that when the polypropylene mesh testicular prosthesis was implanted into the albuginea in the testis, the testicular prosthesis and tissue exhibited an embedded combination. This exhibited good histocompatibility, no prosthesis infection, other complications and adverse reactions occurred after surgery, and no local discomfort and other problems appeared. Compared with traditional orchidectomy, the risk of the operation did not increase. This was consistent with the results of the study conducted by Robinson R et al. [19]. Due to the presence of the testicular prosthesis, the appearance of the scrotum was normal after the operation; hence, the pride of patients was not hurt. Differences in the size and rigidity of the “testis” did not affect the psychological feeling of the patient. The postoperative evaluation of surgical results from patients and their family members were all high, and they were willing to recommend this surgical procedure to their friends. The scores of patients in the experiment group at 3 months and 12 months after surgery did not show a significant change. This suggests that the polypropylene mesh testicular prosthesis continued to maintain a relatively safe and effective character with time after implantation.

Retaining the normal scrotum appearance produced a positive comforting effect for the emotional function of the patient; hence, the overall quality of life of patients was improved. In addition, a study found that good emotional control could have a positive effect on the prognosis of cancer patients [20].

## Conclusions

Therefore, the implantation of polypropylene mesh testicular prosthesis did not increase the risk of surgery and could significantly improve the overall living conditions of patients with advanced prostate cancer. The application of polypropylene mesh testicular prosthesis not only enriches the types of testicular prosthesis, but also promotes the development of testicular prosthesis implantation in patients with prostate cancer. Patients with economic difficulties did not need to bear the high cost of endocrine therapy, which had a greater application value. The author plans to carry out further follow-up studies, in order to promote this self-designed polypropylene mesh testicular prosthesis as a real medical product. Furthermore, the author also plans to gradually extend the application of the implantation of the polypropylene mesh testicular prosthesis to anorchia patients induced by other causes.

## Data Availability

All data generated or analyzed during this study are included in this published article [and its supplementary information files].

## Abbreviations

PSA: Prostate-specific antigen
